# Proteomic analysis and experimental validation reveal the blood–brain barrier protective of Huanshaodan in the treatment of SAMP8 mouse model of Alzheimer’s disease

**DOI:** 10.1186/s13020-024-01016-7

**Published:** 2024-10-05

**Authors:** Yunfang Su, Ningning Liu, Pan Wang, Congcong Shang, Ruiqin Sun, Jinlian Ma, Zhonghua Li, Huifen Ma, Yiran Sun, Zijuan Zhang, Junying Song, Zhishen Xie, Jiangyan Xu, Zhenqiang Zhang

**Affiliations:** 1 Collaborative Innovation Center of Research and Development on the Whole Industry Chain of Yu-Yao, Henan Province; Academy of Chinese Medical Sciences, Henan University of Chinese Medicine, No. 156, Jinshuidong Road, Zhengzhou, 450046 China; 2 The First Affiliated Hospital of Henan University of Chinese Medicine, No. 19, Renmin Road, Zhengzhou, 450046 China; 3 School of Basic Medical Sciences, Henan University of Chinese Medicine, No. 156, Jinshuidong Road, Zhengzhou, 450046 China

**Keywords:** Huanshaodan, Alzheimer’s disease, SAMP8 mice, Proteomic, Fibrinogen

## Abstract

**Background:**

Huanshaodan (HSD) is a Chinese Herbal Compound which has a definite clinical effect on Alzheimer’s disease (AD), however, the underlying mechanism remains unclear. The aim of this study is to preliminarily reveal the mechanism of HSD in the treatment of AD model of SAMP8 mice.

**Methods:**

Chemical composition of HSD and its drug-containing serum were identified by Q-Orbitrap high resolution liquid mass spectrometry. Six-month-old SAMP8 mice were treated with HSD and Donepezil hydrochloride by gavage for 2 months, and Wogonin for 28 days. Behavioral test was performed to test the learning and memory ability of mice. Immunofluorescence (IF) or Western-blot methods were used to detect the levels of p^Ser404^-tau and β-amyloid (Aβ) in the brain of mice. Hematoxylin–eosin (H&E) staining and Transmission electron microscopy (TEM) assay was applied to observe the pathological changes of neurons. Proteomic technology was carried out to analyze and identify the protein network of HSD interventions in AD. Then the pathological process of the revealed AD-related differential proteins was investigated by IF, Q-PCR, Western-blot, Fluorescence in situ hybridization (FISH) and 16S rRNA sequencing methods.

**Results:**

The results showed that HSD and Wogonin, one of the components in its drug-containing serum, can effectively improve the cognitive impairments of SAMP8 mice, protect hippocampal neurons and synapses, and reduce the expression of p^Ser404^-tau and Aβ. HSD and Wogonin reduced the levels of fibrinogen β chain (FGB) and γ chain (FGG), the potential therapeutic targets revealed by proteomics analysis, reduced the colocalization of FGB and FGG with Aβ, ionized calcium binding adaptor molecule 1 (Iba-1), glial fibrillary acidic protein (GFAP), increased level of and myelin basic protein (MBP). Meanwhile, HSD and Wogonin increased ZO-1 and Occludin levels, improved brain microvascular injury, and reduced levels of bacteria/bacterial DNA and lipopolysaccharide (LPS) in the brain of mice. In addition, 16S rRNA sequencing indicated that HSD regulated the structure of intestinal microbiota of mice.

**Conclusion:**

The effects of HSD on AD may be achieved by inhibiting the levels of fibrinogen and the interactions on glia cells in the brain, and by modulating the structure of intestinal microbiota and improving the blood–brain barrier function.

**Supplementary Information:**

The online version contains supplementary material available at 10.1186/s13020-024-01016-7.

## Background

Alzheimer’s disease (AD) is characterized by progressive dementia such as memory loss, cognitive impairment, and personality changes. The incidence of AD is increasing every year as the population grows and ages [1]. The worldwide prevalence of AD will triple by 2050 [2], and the resulting economic costs and social burden are progressive [3]. Amyloid β-protein (Aβ) deposition in the brain is the main pathological feature of AD, which also encompasses intracellular neurofibrillary tangles induced by hyperphosphorylation of tau proteins, as well as progressive neuronal loss [4, 5]. The pathogenesis of AD is highly complex, which makes it difficult to explore drugs against AD. Both genetic factors and environmental toxicants [6] are common causes of AD. Currently, the treatment of AD is limited in clinic [7]. Common anti-AD drugs include donepezil hydrochloride, galantamine, and memantine, as well as the recently marketed gut microbiota modulator GV-971 and the Aβ inhibitor aducanumab, however, the drugs above can only relieve the symptoms to a certain extent, but cannot prevent the progression of the disease [8], even remains controversial [9]. There is evidence that AD is closely related to intestinal function changes [10]. Meanwhile, recent study has confirmed the changes of blood–brain barrier (BBB) in AD, which provides a new way for the diagnosis and treatment of the disease [11]. Currently, AD is seriously jeopardizing human health globally and hindering the world's socio-economic development. Therefore, there is an urgent need to explore new strategies to combat AD [12].

In traditional Chinese medicine, AD belongs to the category of “dementia”, which is closely related to spleen and kidney dysfunction. It is considered that Spleen and kidney deficiency is one of the pathogenesises of dementia [13]. Natural products derived from traditional Chinese medicine include plant-derived natural products and their derivatives, which have neuroprotective effects and may hold promise for the treatment and prevention of AD [14–16]. Huanshaodan (HSD) is derived from Hong Shi Ji Yan Fang in the Song Dynasty of China. It is composed of yam, Achyranthes bidentata, Poria cocos, cornus officinalis, Eucommia Eutria, Broussonetia fructus, Morinda officinalis, Schisandra Chinensis, Lycium barbarum, ripened ground, Cistanche tubulosa, Polygala tenuifolia, Acorus tatarinowii, Foeniculum vulgare and jujube. HSD is a prescription recommended by the Chinese Medicine Joint Consensus Group on the diagnosis and treatment of AD [17] with definite clinical efficacy [18]. However, few molecular mechanisms have been reported for the treatment of AD by HSD.

SAMP8 mice are rapidly aging mice which belongs to physiological kidney deficiency syndrome [19]. SAMP8 mice show typical pathological features associated with AD, such as age-dependent learning and memory decline, Aβ plaque deposition, tau protein hyperphosphorylation, oxidative stress, glial cell degeneration, neuroinflammatory response, synaptic changes and neuronal degeneration loss during the growth process, which is a commonly used animal model for AD research [20]. The chemical components in HSD and its drug-containing serum was identified by LC–MS/MS. The pharmacological effect of HSD and Wogonin, which is one of its components in drug-containing serum, were confirmed in SAMP8 mice. In order to find out the therapeutic mechanism of HSD in AD, proteomics TMT protein quantitative technology was performed to analyze the protein network of HSD intervention in SAMP8 mice. The differential protein expression profiles and key molecular targets and related pathological processes of HSD were identified and investigated in the improvement of AD.

## Materials and methods

### Drugs and reagents

HSD (State Food and Drug Administration (SFDA) approval number: Z20025017, Chongqing Taiji Group TongJunGe Pharmaceutical Co., LTD, China); Donepezil hydrochloride (SFDA approval number: H20010723, Weicai Pharmaceutical Co., LTD, China); Wogonin (Lot: A0425C, meilune, Dalian). Details of antibodies are shown in Table 1.

**Table 1:** Antibodies used in this study

Antibodies (Catalog number)	Source	Application/Dilution
Actin (BM0627)	BOSTER	WB/1:5000
p^Ser404^-tau (20194S)	Cell Signaling Technology	WB/1:1000; IF/1:500
Aβ_1-42_ (ab201060)	abcam	IF/1:200; IHC/1:500
Occludin (GB111401)	Servicebio	WB/1:1000
ZO-1 (GB111402)	Servicebio	WB/1:1000
FGB (ab189490)	abcam	WB/1:1000; IF/1:1000
FGG (ab281924)	abcam	WB/1:1000; IF/1:1000
Syn (5297S)	Cell Signaling Technology	WB/1:1000
GFAP (12389S)	Cell Signaling Technology	IF/1:400
Iba-1 (GB113502)	Servicebio	IF/1:500
MBP (GB12226)	Servicebio	IF/1:500
LPS (ab35654)	abcam	IF/1:200
Horseradish peroxidase (HRP)-linked secondary antibodies against mouse IgG of the primary antibodies (ZB-2305)	ZSGB-BIO	WB/1:8000
Horseradish peroxidase (HRP)-linked secondary antibodies against rabbit IgG of the primary antibodies (ZB-2301)	ZSGB-BIO	WB/1:8000
HRP Polyclonal Goat Anti-Rabbit IgG (GB23303)	Servicebio	IF/1:500
Alexa Fluor 488-labeled goat anti-mouse IgG (GB25301)	Servicebio	IF/1:400
CY3-Tyramide (TSA) (G1223)	Servicebio	IF/1:500

### Preparation and component analysis of HSD medicated serum

The drug-containing serum of HSD was prepared using male KM mice of (40 ± 5) g weight which were obtained from Beijing Vital River Laboratory Animal Technology Co., LTD. (license number was SCXK (zhe) 2020-0002). Animals were kept in animal laboratory center. Experiment began after one week of adaptive feeding. Dissolved HSD in sterile 0.9% saline at a dose of 5.4 g/kg/day, and mice were administered by gavage for 7 days. Following the last gavage, blood samples were collected by enucleation 3 h (fasting for 12 h before blood collection), serum was routinely collected and inactivated, and finally stored in a −80 °C refrigerator. The experimental procedures were carried out according to the principles of experimental animal welfare (Ethical approval number of experimental animals: DWLLGZR202202147).

Appropriate amount of HSD pills and drug-containing serum were taken for component analysis. An appropriate amount of 80% methanol solution was added to the samples, ground thoroughly, vortexed and centrifuged to extract the supernatant for analysis. Q-Orbitrap high-resolution liquid chromatography mass spectrometry was used to identify the chemical composition of the drug (Servicebio, project number: CD019). CD2.1 (Thermo Fisher) software was used for preliminary sequencing of the high-resolution liquid acquisition data, which were then searched and compared in a database of mzCloud.

### Animals and treatment

Six-month-old male SAMP8 and SAMR1 were obtained from Beijing Vital River Laboratory Animal Technology Co., LTD. (license number was SCXK (jing) 2016-0010). Rearing and ethical conditions were the same as those described above for KM mice. The mice were randomly divided into 4 groups: control group, model group, donepezil group (0.747 mg/kg/day), HSD group (2.7 g/kg/day, based on our previous study [21] and Wogonin group (40 mg/kg/day, adjusted based on the reports [22]) with 12 mice in each group. HSD and donepezil were dissolved in 0.9% saline and Wogonin was suspended in 0.5% sodium carboxymethyl cellulose. Mice in Donepezil and HSD groups were gavaged for 2 months, and Wogonin group was given for 28 days. A same volume of 0.9% saline was given to the mice in control and model groups.

### Behavioral tests

Morris water maze, open field and Y-maze test, which are commonly used for behavioral tests in AD, were performed during the last 6 days of the experiment. The detailed procedures were described in our previous publication [23].

### Hematoxylin–eosin (H&E) staining

After the behavioral experiments, mice were anaesthetized with isoflurane and brain tissues were collected in 4% paraformaldehyde solution. 5 μm of paraffin sections were made, and H&E staining was performed by conventional methods. Tissue images were observed and photographed using a microscope (Axioscope 5, ZEISS, Germany).

### Western blotting

The protein levels of p^Ser404^-tau, FGB, FGG, ZO-1 and Occludin were detected by conventional Western-blotting assay which were described in our previous study [24].

### Real-time quantitative PCR (RT-qpcr) assay

An appropriate amount of hippocampal or cortical tissue was added to 1 mL TRIzol. RNA was routinely extracted using RNA extraction kit. RNA concentration was tested by Nanodrop spectrophotometer and reverse transcription was performed using Vazyme reverse transcription kit. Q-PCR was run using a Real-time PCR instrument (Roche, Switzerland). The primers are shown in Table 2.

**Table 2:** Primer sequences of gene-specific primers used for qPCR

Primer name	Primer sequence (5ʹ–3ʹ)
β-Actin F	GGCTGTATTCCCCTCCATCG
β-Actin R	CCAGTTGGTAACAATGCCATGT
Occludin F	ACACTTGCTTGGGACAGAGG
Occludin R	AAGGAAGCGATGAAGCAGAA
ZO-1 F	GACCTTGATTTGCATGACGA
ZO-1 R	AGGACCGTGTAATGGCAGAC

### IF staining

Paraffin sections prepared in H&E staining were dewaxed with xylene, sealed with 3% H_2_O_2_, repaired with sodium citrate antigen, and sealed with 5% goat serum, p^Ser404^-tau/LPS and HRP Polyclonal Goat Anti-Rabbit IgG were used as the primary and secondary antibody, respectively. For the immunofluorescence co-localization, after the completion of immunofluorescence single labeled secondary antibody incubation, the slices were washed 3 times in PBS for 5 min each time. After slightly drying, TSA was added dropwise and incubated in dark at room temperature for 10 min. Then, the slices were washed 3 times in TBST for 5 min each time, followed by antigen repair and the addition of the second primary antibody. Finally, nuclear staining was carried out using DAPI. A microscopewas used to observe and photograph of the tissues (Axioscope 5, ZEISS, Germany).

### IHC staining

Aβ_1-42_ antibody was used as the primary antibody for routine immunohistochemical staining performed on paraffin sections. Images were taken under microscope, and the average optical density values of Aβ_1-42_ were calculated by ImagePro Plus software.

### TEM assay

Hippocampal tissues of appropriate size were fixed with 2.5% glutaraldehyde, conventional TEM assay which were described in our previous study [25] were performed. Samples were taken and observed by a projection electron microscope (HT7800, HITACHI, Japan) and photographed. The ultrastructure of neurons, synapses and blood vessels were observed. TEM assay was carried out by TEM Center of Henan University of Chinese Medicine.

### Proteomics analysis

The mouse hippocampus tissue in pharmacodynamic study was used for proteomics analysis through TMT™ (Tandem Mass Tag™) method. Firstly, the quality control of protein extraction was performed, and the samples were labeled after enzymatic salt removal. Secondly, the mixed labeled samples were processed by high performance liquid chromatography (HPLC), and finally analyzed by mass spectrometry. The proteomics part of the experiment was jointly completed by Suzhou PANOMIX Biomedical Tech Co., LTD. (project number: HT2022061813001).

### Fluorescence in situ hybridization (FISH) assay

A universal fluorescent probe EUB338 [26] targeting 16S rRNA was used to detect the bacterial specific DNA fragments in the paraffin-embedded brain sections of mice in each group by FISH method. After deparaffinization to water, the paraffin sections were digested, hybridized, and finally stained for nuclei with DAPI. Images were observed and taken under a microscope (Axioscope 5, ZEISS, Germany).

### 16s rrna analysis

From each group, five mice were randomly selected for collection of cecum contents. The microbiota in cecal contents was analyzed by conventional 16S rRNA sequencing. Alpha diversity analysis (including Simpson and Goods_coverage index) and Beta diversity analysis (Bray–Curtis based NMDS, with hull) were carried out. Heat maps for species composition in genus-level were drawn. LEfSe analysis were performed [27]. The abundance at the phylum to genus levels among each group was statistically analyzed. 16S rRNA gene sequencing was commissioned by Suzhou PANOMIX Biomedical Tech Co., LTD. (project number: HT2022061813001). Specific experimental methods are described in detail in our previous study [23].

### Statistical analysis

The statistical analysis was performed using GraphPad Prism software. The escape latency of behavioral experiments was analyzed by two-way ANOVA, and the Western-blot data were analyzed by one-way ANOVA. Data were expressed as Mean ± standard error (Mean ± SEM). In proteomics, T-test method was used for difference analysis, *P* value of <0.05, and a fold change of 1.2 was considered to be significant difference.

## Results

### Analysis of compounds in HSD and its drug-containing serum

Figure S1 shows the total ion chromatogram for the identification of the compounds in the HSD and its drug-containing serum. The chemical composition of HSD matched 237 compounds with a composite score greater than 60 in mzCloud best match, and 30 small molecular compounds of traditional Chinese medicine were screened by comparison with the traditional Chinese medicine database (Shown in Table S1). While the chemical composition of HSD drug-containing serum full matched 61 small molecular compounds in mzCloud best match (Shown in Table S2). Among them, Wogonin identified both in HSD and in its drug-containing serum was selected as a drug in the subsequent experimental study.

### HSD improves cognitive disorder in samp8 mice

**Fig. 1:**
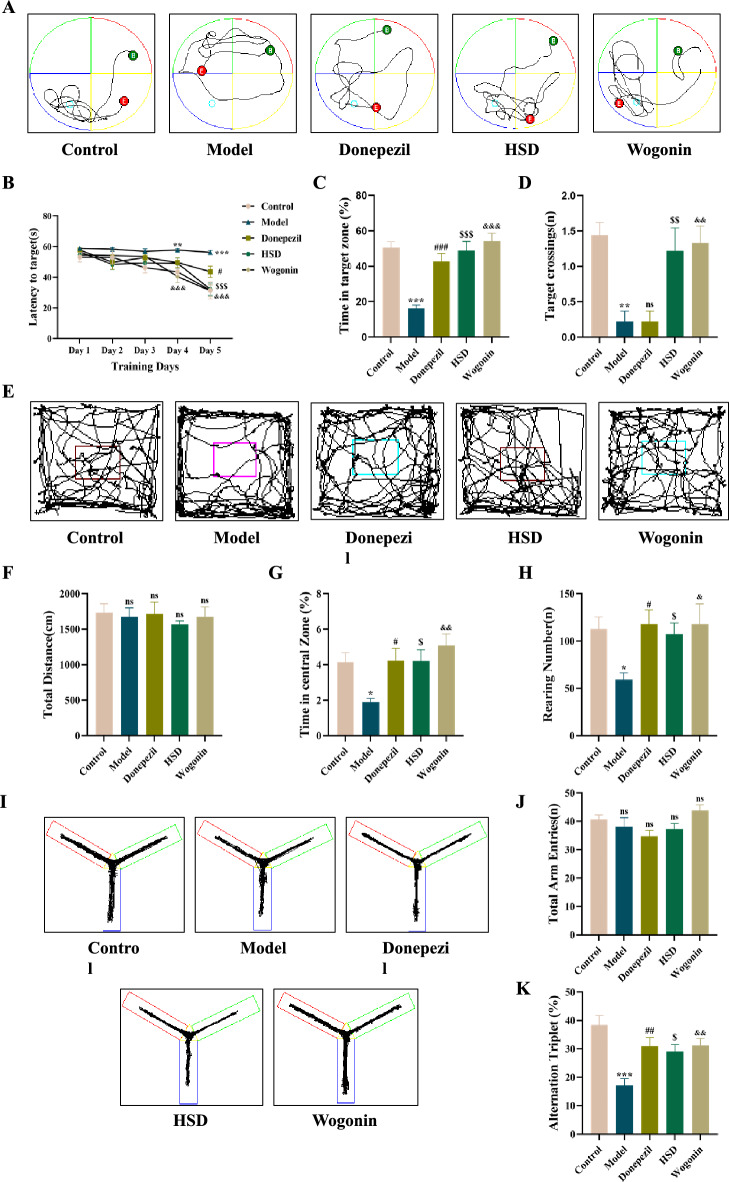
Behavioral experiments. **A** Swimming trajectories of the Morris water maze experiment; **B** Evasion latency; **C** Time elapsed in target quadrant; **D** Platform crossings times; **E** Activity trajectories of the open field experiment; **F** Total distance; **G** Percentage of time in central zone; **H** Number of grooming; **I** Activity trajectory of the Y maze experiment; **J** Arm entries times; **K** Spontaneous alternating correct rate of Y maze in each group. The data are expressed as Mean ± SEM (*n* = 9). *: model group compared with the control group, #: Donepezil group compared with the model group, $: HSD group compared with the model group, &: Wogonin group compared with the model group. *ns* no significant difference, *^,#,$,&^*P* < 0.05, **^,##,$$,&&^*P* < 0.01, ***^,###,$$$,&&&^*P* < 0.001

The result of Morris water maze test showed that the escape latency of mice was significantly prolonged in the model group (*P* < 0.01) from the 4th day comparing with those in the control group. On the 5th day, the escape latency of mice in the donepezil group, HSD group and Wogonin group were significantly shortened comparing with the model group (*P* < 0.05, *P* < 0.001, *P* < 0.001) (Fig. 1B). On the last day of the space exploration experiment, the number of platform crossings and the time spent in the target quadrant of mice in the model group were significantly lower than those in the control group (*P* < 0.001, *P* < 0.01) (Fig. 1C, D). The donepezil group, HSD group and Wogonin group had significant increases in the time spent in the target quadrant compared with the model group (*P* < 0.001, *P* < 0.001, *P* < 0.001) (Fig. 1C). There was no significant difference in the number of platform crossings between the donepezil group and the model group, but the number of platform crossings in the HSD group and Wogonin group were significantly higher than that in the model group (*P* < 0.01, *P* < 0.01) (Fig. 1D). The open field results showed that there was no significant difference in the total moving distance among the groups (Fig. 1F). Compared with the control group, the time spent in the central area and the times of grooming in the model group were significantly reduced (*P* < 0.05, *P* < 0.05). Compared with the model group, the time spent in the central area and the times of grooming in the donepezil and HSD group were significantly increased (*P* < 0.05, *P* < 0.05, *P* < 0.01; *P* < 0.05, *P* < 0.05, *P* < 0.05) (Fig. 1G, H). The results of Y maze showed that there was no significant difference in the total number of arm approaches among the three groups (Fig. 1J). Compared with the control group, the model group had a significant reduction in the rate of arm entry altercountry (*P* < 0.001). Compared with the model group, the donepezil group and HSD group had a significant increase in the rate of arm entry altercountry (*P* < 0.01, *P* < 0.05) (Fig. 1K).

### HSD reduced the protein levels of p^Ser404^-Tau and Aβ and the histopathological changes of the brain tissue in SAMP8 mice

**Fig. 2:**
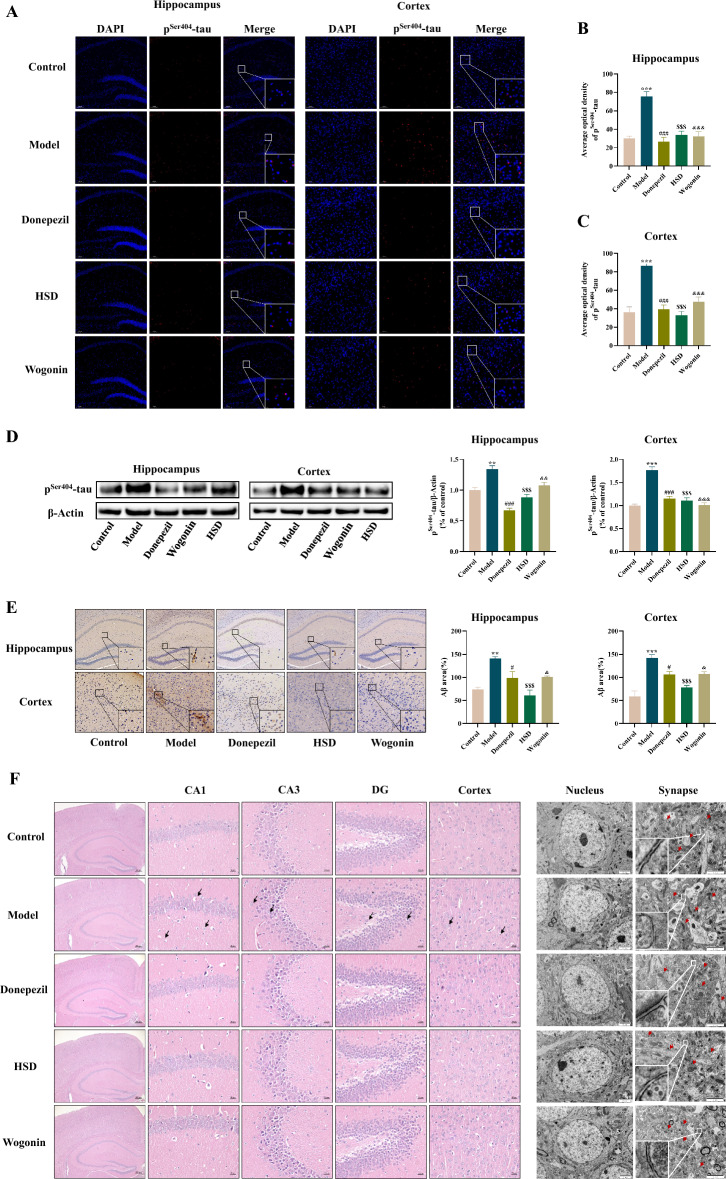
The expression levels of p^Ser404^-tau and Aβ in the hippocampus and cortex and morphological structure of neurons and synapses observed by H&E staining and transmission electron microscopy. **A** Fluorescence expression of p^Ser404^-tau in hippocampus (*left*) and cortex (*right*) of mice in each group. p^Ser404^-tau protein staining (*red*) and nuclei re-stained (*blue*) (Magnification 100×, partial enlarged is Magnification 1000×). **B** The mean density of p^Ser404^-tau fluorescence in hippocampus of mice in each group. **C** Mean density analysis of p^Ser404^-tau fluorescence in cortex of mice in each group; **D** Expression of p^Ser404^-tau protein in hippocampus and cortex of mice in each group; **E** Aβ deposition in hippocampus and cortex of mice in each group (Magnification 200×, partial enlarged is Magnification 1000×); **F** H&E staining (Magnification 40×, partial enlarged is Magnification 400×, neuronal cells were loosely arranged with karyopyknosis in model group, shown with *black arrow*) and transmission electron microscopy (Magnification 20,000×, red arrows indicating synaptic structure). The data are expressed as Mean ± SEM (*n* = 3). *: model group compared with the control group, #: Donepezil group compared with the model group, $: HSD group compared with the model group, &: Wogonin group compared with the model group. *^,#,$,&^*P* < 0.05, **^,##,$$,&&^*P* < 0.01, ***^,###,$$$,&&&^*P* < 0.001

Immunofluorescence results showed that the protein levels of p^Ser404^-tau in the hippocampus of the model group was significantly higher than that of in the control group (*P* < 0.001), the levels of p^Ser404^-tau in hippocampus of donepezil group, HSD group and Wogonin group was significantly lower than that of model group (*P* < 0.001, *P* < 0.001, *P* < 0.001) (Fig. 2B); Compared with the control group, the model group had a significant increase in the expression of p^Ser404^-tau in the cortex (*P* < 0.001), and the donepezil, HSD and Wogonin groups had significant reduction in the expression of p^Ser404^-tau in the cortex compared with the model group (*P* < 0.001, *P* < 0.001, *P* < 0.001) (Fig. 2C); Similarly, Western-blot results showed that the expression of p^Ser404^-tau protein in the hippocampus of the model group was significantly higher than that of the control group (*P* < 0.01), while donepezil, HSD and Wogonin significantly reduced the levels of p^Ser404^-tau protein in the hippocampal tissues (*P* < 0.001, *P* < 0.001, *P* < 0.01); The expression of p^Ser404^-tau protein in the cortex of the model group was significantly higher than that in the control group (*P* < 0.001), while donepezil, HSD and Wogonin significantly reduced the expression of p^Ser404^-tau protein in the cortical tissues (*P* < 0.001, *P* < 0.001, *P* < 0.001) (Fig. 2D).

The immunohistochemistry results showed that the Aβ deposition in the hippocampus and cortex of the model group was significantly higher than that of the control group (*P* < 0.01, *P* < 0.001). Compared with the model group, the Aβ deposition in the hippocampus and cortex of the donepezil group was significantly reduced (*P* < 0.05, *P* < 0.05). The deposition of Aβ in hippocampus and cortex was significantly reduced in HSD group (*P* < 0.001, *P* < 0.001), and the deposition of Aβ in hippocampus and cortex was also significantly reduced in Wogonin group (*P* < 0.05, *P* < 0.05) (Fig. 2E).

The results of H&E staining showed that the neurons in the hippocampus CA1, CA3, dentate gyrus and cortex of the control group were structurally intact, neatly arranged, and the nuclei were clearly stained, while the neurons in the hippocampus and cortex of the model group showed neuropathological changes such as the reduction in the number of neurons, loose arrangement, cell vacuoles, and nuclear pyknosis. The pathological changes of hippocampus and cortex in donepezil group, HSD group and Wogonin group were improved, the number of nerve cells increased, the arrangement of nerve cells was more regular, and the cell vacuolization and karyopyknosis were less (Fig. 2F). Transmission electron microscopy showed that the nucleus of the hippocampal CA1 region was atrophic and deformed, the structure of synaptic membrane was incomplete, the synaptic cleft was mostly blurred, and the vesicles were sparse in the model group. In the control group, donepezil group, HSD group and Wogonin group, the nucleus showed normal with clear synaptic structure and synaptic gap (Fig. 2F).

### Proteomics analysis of hippocampal tissue of samp8 mice

**Fig. 3:**
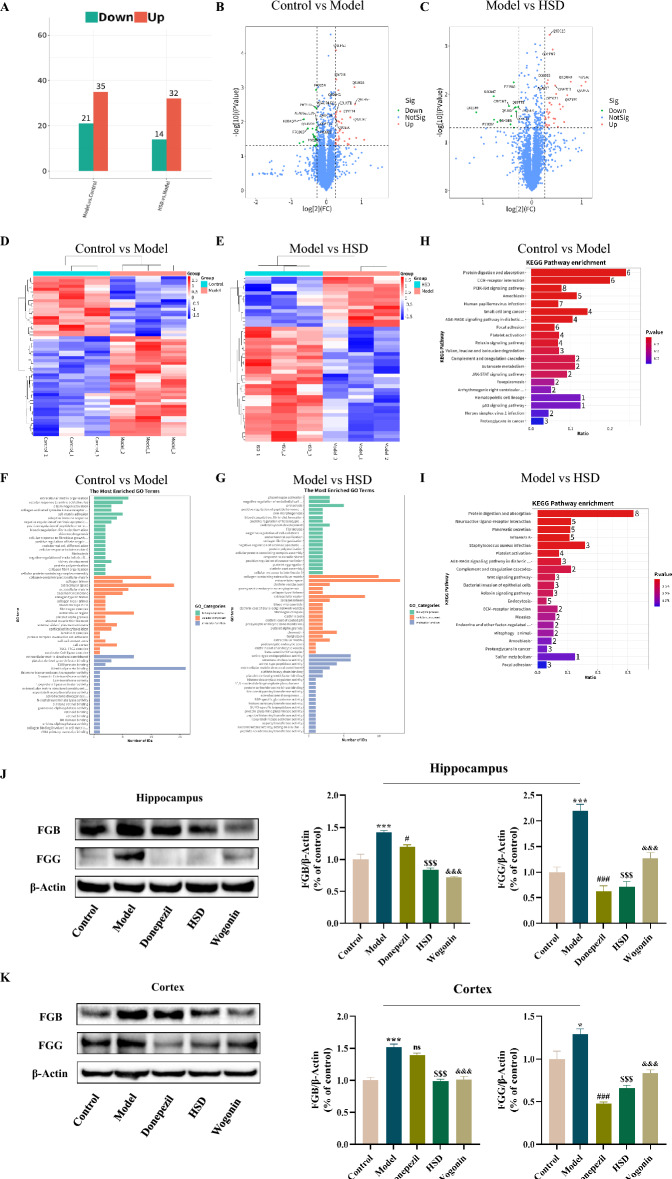
Proteomics analysis of hippocampus tissue of SAMP8 mice treated with HSD and the expression levels of FGB and FGG in the brain of mice in each group. **A** Number of differentially expressed proteins; **B**, **C** Volcano plot statistics of differentially expressed proteins in control group vs. model group and model group vs. HSD group, respectively; **D**, **E** Heat map statistics of differentially expressed proteins in control group vs. model group and model group vs. HSD group, respectively; **F**, **G** GO enrichment analysis of differentially expressed proteins in control group vs. model group and model group vs. HSD group, respectively; **H**, **I** KEGG pathway enrichment analysis of differentially expressed proteins in control group vs. model group and model group vs. HSD group, respectively; **J**, **K** FGB and FGG protein expression levels in hippocampus and cortex of mice in each group, respectively. The data are expressed as Mean ± SEM (*n* = 3). *: model group compared with the control group, #: Donepezil group compared with the model group, $: HSD group compared with the model group, &: Wogonin group compared with the model group. ns no significant difference, *^,#,$,&^*P* < 0.05, **^,##,$$,&&^*P* < 0.01, ***^,###,$$$,&&&^*P* < 0.001

A total of 56 differentially expressed proteins were obtained from the control group and the model group (more than 1.2 times as significant difference), in which 35 proteins were up-regulated with 21 proteins were down-regulated. A total of 46 differentially expressed proteins were obtained from the model group and the HSD group, of which 32 proteins were up-regulated with 14 proteins were down-regulated, while 12 proteins were differentially expressed in the three groups (Fig. 3A). Among them, 9 proteins were down-regulated and 3 proteins were up-regulated by HSD (Table S3). The volcano plot reflected the difference of the overall protein. The differentially expressed proteins were screened by FC < 1/1.2 and FC > 1.2, and *P* < 0.05. The red dots in the upper right corner represent up-regulated differentially expressed proteins, and the green dots in the upper left corner represent down-regulated differentially expressed proteins (Fig. 3B, C). The differential protein cluster heatmap intuitively reflected the differential expression changes of differential proteins in the two groups. Hierarchical clustering was performed on the expression patterns of differential proteins, and the clustering results were presented using a heat map. The darker the red color, the higher the protein expression. The darker the blue color, the lower the expression of the protein (Fig. 3D, E). GO functional enrichment analysis was performed on the differentially expressed proteins. A total of 453 items were enriched in Biological Process (BP) and 152 items were enriched in Cellular Component (CC) in the control group and the model group, respectively. A total of 112 items were enriched for Molecular Function (MF); In the model group and HSD group, a total of 323 items were enriched in BP, 97 items were enriched in CC, and 117 items were enriched in MF. Each subclass was annotated according to the top 20 most-enriched GO enriched terms. According to the annotation results, the GO function of the control group, model group and HSD group was mainly involved in extracellular matrix, fibrin and plasminogen, collagen, endothelial cell apoptosis, platelet regulation, etc. (Fig. 3F, G). According to the differentially expressed proteins, KEGG pathway enrichment analysis was performed, and 83 pathways were enriched in the control group and the model group, and 102 pathways were enriched in the model group and HSD group. Annotation was performed according to the top 20 most enriched KEGG pathways. The KEGG pathways of the control group, model group and HSD group were mainly involved in the Extracellular matrix (ECM) interaction of the central nervous system, complement and coagulation cascade, platelet activation, etc. (Fig. 3H, I).

Western-blot results showed that the model group had significant increases in the protein expression levels of Fibrinogen beta chain (FGB) and Fibrinogen γ chain (FGG) in the hippocampus compared with the control group (*P* < 0.001, *P* < 0.001), and the donepezil group had significant reductions in the protein expression levels of FGB and FGG in the hippocampus compared with the model group (*P* < 0.05, *P* < 0.001), meanwhile, the expressions of FGB and FGG in hippocampus were significantly decreased in HSD group (*P* < 0.001, *P* < 0.001) and Wogonin group (*P* < 0.001, *P* < 0.001) (Fig. 3J). Compared with the control group, the model group had significant increases in the protein expression levels of FGB and FGG in the cortex tissue (*P* < 0.001, *P* < 0.05). Compared with the model group, the donepezil group had no significant difference in the expression of FGB in the cortex tissue, and the expression of FGG was significantly decreased (*P* < 0.001). The expression of FGB and FGG in cortex tissue was significantly decreased in HSD group (*P* < 0.001, *P* < 0.001), while it was also significantly decreased in Wogonin group (*P* < 0.001, *P* < 0.001) (Fig. 3K).

### HSD reduced the co-localization of FG and Aβ in brain of samp8 mice

**Fig. 4:**
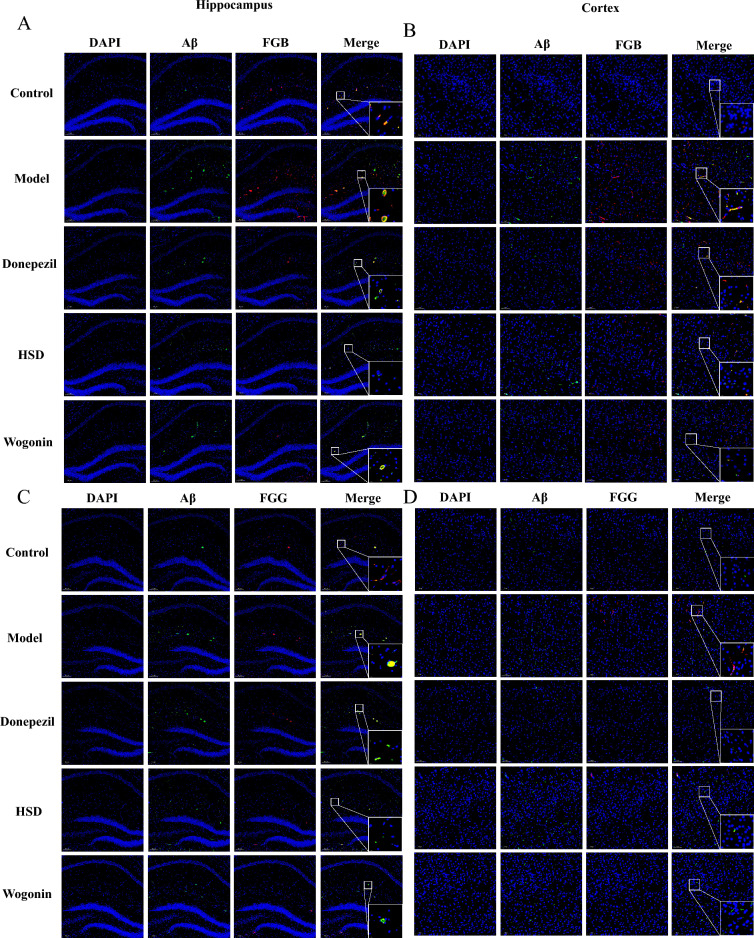
Colocalization of FGB, FGG and Aβ in hippocampus and cortex of mice in each group (Magnification 100×, partial enlarged is Magnification 1000×). **A**, **B** Colocalization of FGB and Aβ in hippocampus and cortex; **C**, **D** Colocalization of FGG and Aβ in hippocampus and cortex. *Blue*, *green* and *red* fluorescence indicates DAPI nuclear staining, Aβ and FGB/FGG, respectively

The results of immunofluorescence showed that the positive expression of FGB and Aβ were increased obviously with a colocalization in the model group comparing with the control group, while the donepezil group, HSD group and Wogonin group had reduced the expression of FGB and Aβ, and the aggregation of FGB and Aβ (Fig. 4A, B). Similarly, compared with the control group, the model group had an obvious increase in the expression of FGG and Aβ with a colocalization, while the donepezil group, HSD group and Wogonin group had a reduction in the expression of FGG and Aβ, and a reduction in the aggregation of FGG and Aβ (Fig. 4C, D).

### HSD reduced the co-localization of FG and iba-1 and inhibited the activation of microglia

**Fig. 5:**
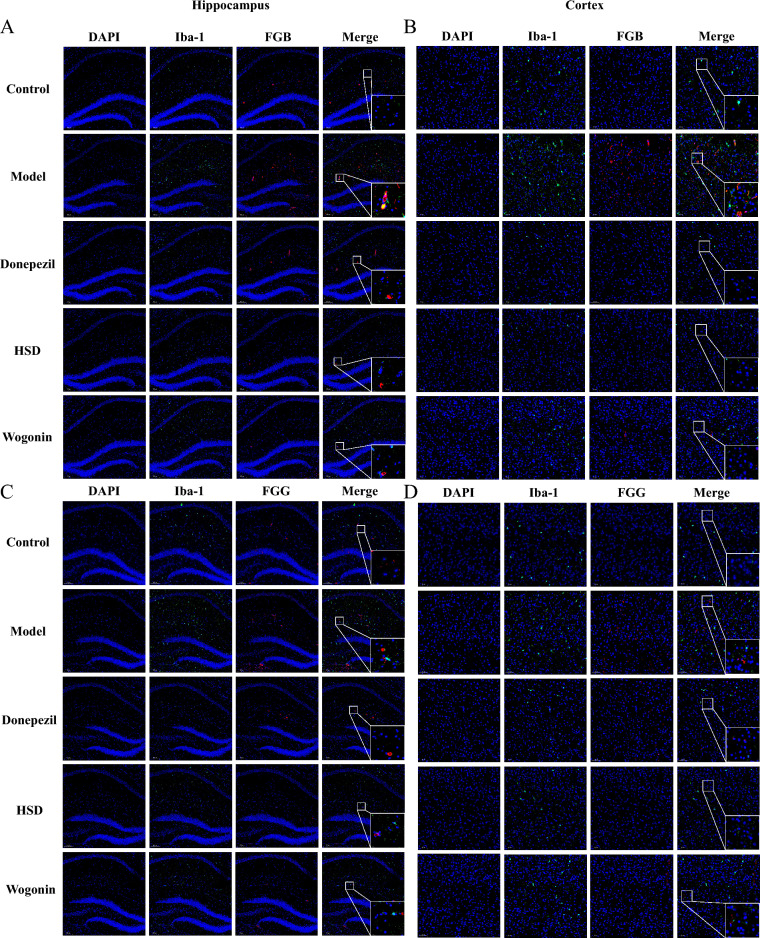
Colocalization of FGB, FGG and Iba-1 in hippocampus and cortex of mice in each group (Magnification 100×, partial enlarged is Magnification 1000×). **A**, **B** Colocalization of FGB and Iba-1 in hippocampus and cortex; **C**, **D** Colocalization of FGG and Iba-1 in hippocampus and cortex. *Blue*, *green* and *red* fluorescence indicates DAPI nuclear staining, Iba-1 and FGB/FGG, respectively

Figure 5 showed the positive expression of FGB and Iba-1 were increased obviously with a colocalization in the model group comparing with the control group, while the donepezil group, HSD group and Wogonin group had reduced the expression of FGB and Iba-1, and the aggregation of FGB and Iba-1 (Fig. 5A, B). Meanwhile, the model group had an obvious increase in the expression of FGG and Iba-1 with a colocalization comparing with the control group, while the donepezil group, HSD group and Wogonin group had a reduction in the expression of FGG and Iba-1, and a reduction in the aggregation of FGG and Iba-1 (Fig. 5C, D).

### HSD reduced the co-localization of FG and GFAP and inhibited the activation of astrocytes

**Fig. 6:**
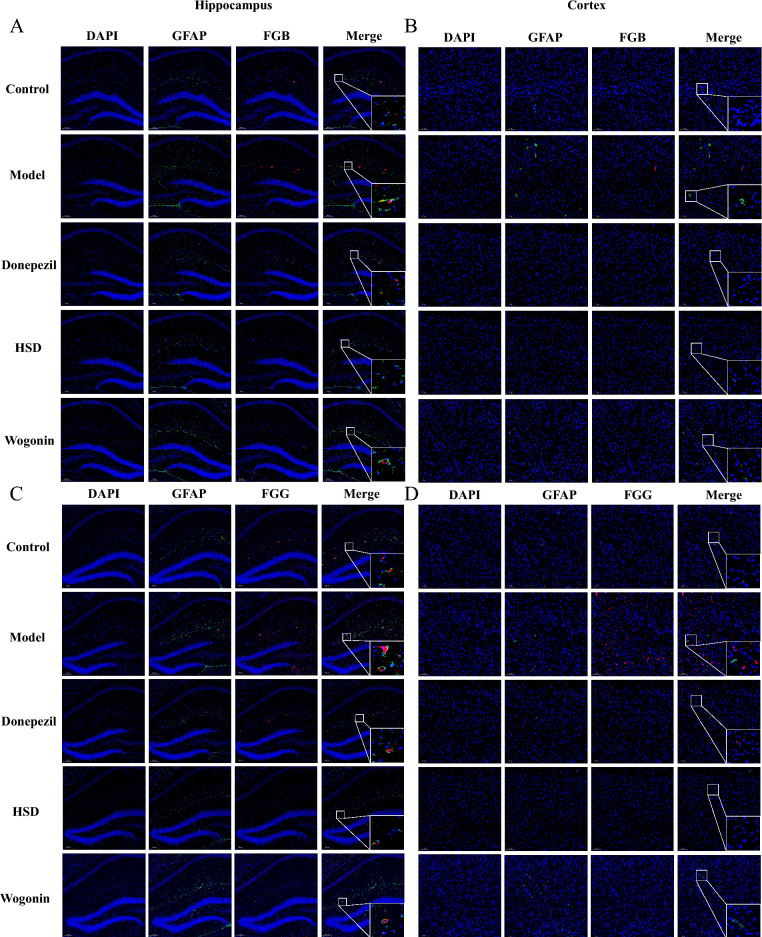
Colocalization of FGB, FGG and GFAP in hippocampus and cortex of mice in each group (Magnification 100×, partial enlarged is Magnification 1000×). **A**, **B** Colocalization of FGB and GFAP in hippocampus and cortex; **C**, **D** Colocalization of FGG and GFAP in hippocampus and cortex. *Blue*, *green* and *red* fluorescence indicates DAPI nuclear staining, GFAP and FGB/FGG, respectively

As shown in Fig. 6, compared with the control group, the model group had an obviously increase in the expression of FGB and GFAP with a colocalization, while the donepezil group, HSD group and Wogonin group had a reduction in the expression of FGB and GFAP, and the aggregation of FGB and GFAP (Fig. 6A, B). Similarly, compared with the control group, the expression of FGG and GFAP in the model group was increased with obvious colocalization, while the expression and aggregation of FGG and GFAP in the donepezil group, HSD group and Wogonin group were reduced (Fig. 6C, D).

### HSD increased myelin generation of oligodendrocytes

**Fig. 7:**
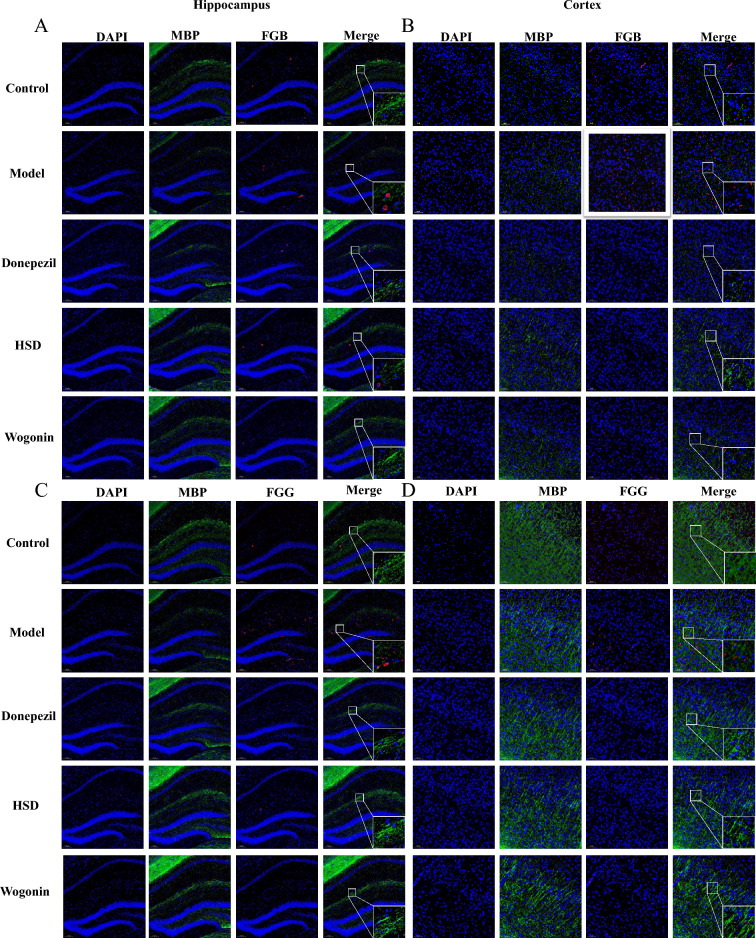
FGB, FGG and MBP in hippocampus and cortex of mice in each group (Magnification 100×, partial enlarged is Magnification 1000×). **A**, **B** Positive expression of FGB and MBP in hippocampus and cortex; **C**, **D** Positive expression of FGG and MBP in hippocampus and cortex. *Blue*, *green* and *red *fluorescence indicates DAPI nuclear staining, MBP and FGB/FGG, respectively

The immunofluorescence results showed that the MBP positive expression in hippocampus and cortex of the model group was lower than that of the control group, while the MBP expression in the donepezil group, HSD group and Wogonin group were higher than that in the model group. It is shown that there was no colocalization of FGB and FGG with MBP (Fig. 7).

### HSD improved the vascular ultrastructure of samp8 mice, increased the protein levels of ZO-1 and occludin in the brain barrier, and reduced the level of bacterial DNA fragments and LPS in the brain

**Fig. 8:**
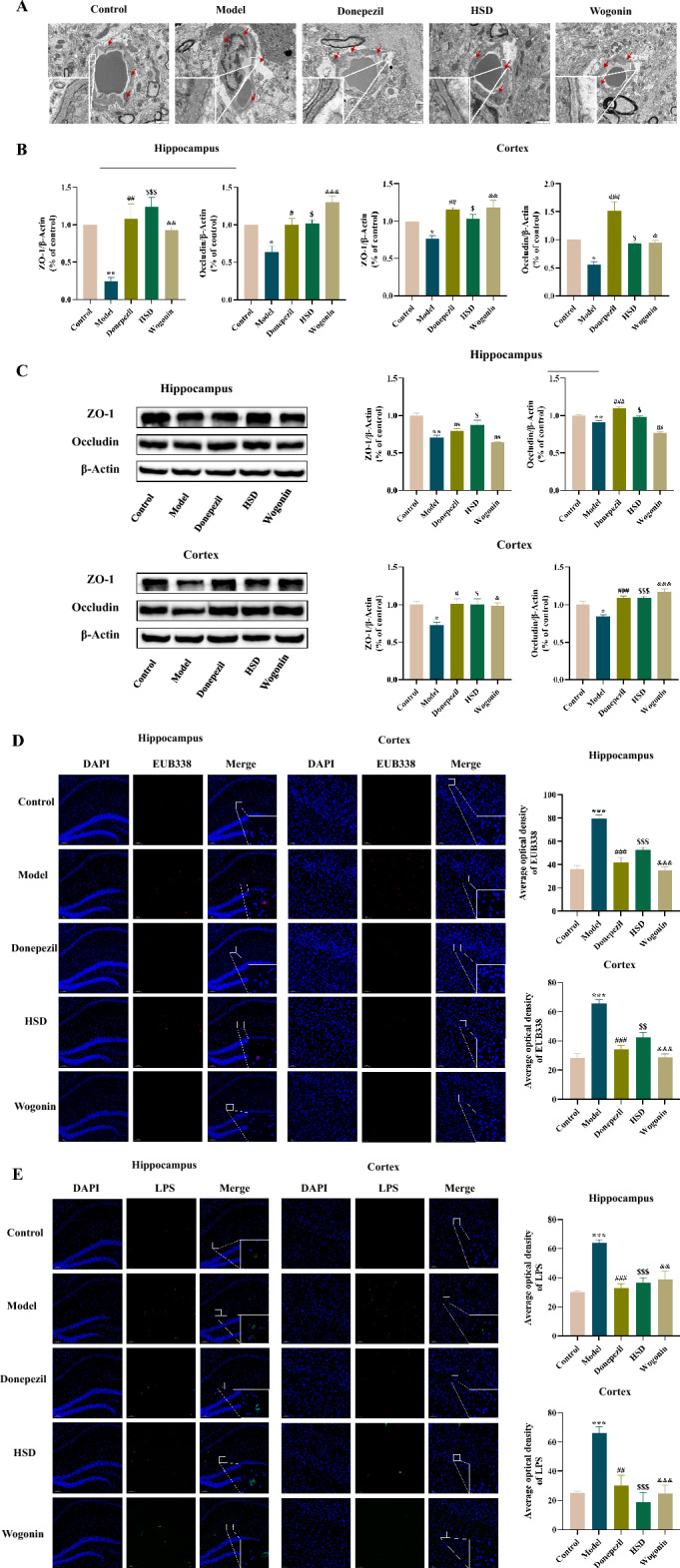
The ultrastructure of brain vessels, the expression levels of ZO-1 and Occludin, the relative content of bacteria or bacterial DNA fragments and LPS in the brain of mice in each group. **A** Ultrastructure of hippocampal blood vessels in each group (Magnification 30,000×), *red scissors* indicated tight junctions between endothelial cells, basement membrane, mitochondria and glial endfeet; **B** The mRNA expression levels of ZO-1 and Occludin in hippocampus and cortex of mice in each group; **C** Protein expression levels of ZO-1 and Occludin in hippocampus and cortex of mice in each group; **D** In situ hybridization of EUB338 fluorescent probe in hippocampus and cortex of mice in each group, *blue* and *red *fluorescence indicates DAPI nuclear staining and EUB338 positive signal, respectively (Magnification 100×, partial enlarged is Magnification 1000×); **E** Immunofluorescence staining of LPS in hippocampus and cortex of mice in each group, blue and green fluorescence indicates DAPI nuclear staining and LPS positive signal, respectively (Magnification 100×, partial enlarged is Magnification 1000×). The data are expressed as Mean ± SEM (*n* = 3). *: model group compared with the control group, #: Donepezil group compared with the model group, $: HSD group compared with the model group, &: Wogonin group compared with the model group. ns no significant difference, *^,#,$,&^*P* < 0.05, **^,##,$$,&&^*P* < 0.01, ***^,###,$$$,&&&^*P* < 0.001

The morphological changes of microvessels in the brain were observed by TEM. The results showed the tight junctions between endothelial cells were blurred, the basement membrane was broken and blurred, the mitochondria of endothelial cells were swollen, the membrane was ruptured, the cristae were mostly dissolved, and the end feet of glial cells were swollen and deformed in the model group. In the donepezil group, HSD group and Wogonin group, the tight junctions between endothelial cells were clear, the basement membrane was tightly connected, and the endothelial mitochondria and glial structures tended to be in a healthy state (Fig. 8A).

The mRNA levels of ZO-1 and Occludin in brain tissue were detected by Q-PCR. The results showed that the expression of ZO-1 mRNA in hippocampus and cortex in the model group was significantly decreased comparing with the control group (*P* < 0.01, *P* < 0.05). Compared with the model group, the expression of ZO-1 mRNA in hippocampus and cortex in the donepezil group was significantly increased (*P* < 0.01, *P* < 0.01), the expression of ZO-1 mRNA in hippocampus and cortex was significantly increased in HSD group (*P* < 0.001, *P* < 0.05), and it was also significantly increased in Wogonin group (*P* < 0.01, *P* < 0.01). The expression of Occludin mRNA in the hippocampus and cortex of the model group was significantly decreased comparing with the control group (*P* < 0.05, *P* < 0.05). Compared with the model group, the expression of Occludin mRNA in the hippocampus and cortex of the donepezil group was significantly increased (*P* < 0.05, *P* < 0.001), it was significantly increased in HSD group (*P* < 0.05, *P* < 0.05) and in Wogonin group (*P* < 0.001, *P* < 0.05) (Fig. 8B). Similar to the Q-PCR results, Western-blot results showed that the expression of ZO-1 in the hippocampus and cortex of the model group was significantly decreased comparing with the control group (*P* < 0.01, *P* < 0.05). Compared with the model group, there was no significant difference in the expression of ZO-1 in the hippocampus of the donepezil group, while the expression of ZO-1 in the cortex was significantly increased (*P* < 0.05), it was also increased in HSD group (*P* < 0.05, *P* < 0.05) and in Wogonin group (*P* < 0.01, *P* < 0.05). Compared with the control group, the expression of Occludin in the hippocampus and cortex of the model group was significantly decreased (*P* < 0.01, *P* < 0.05) while which was significantly increased in donepezil group comparing with the model group (*P* < 0.001, *P* < 0.001), meanwhile, the expression of Occludin in the hippocampus and cortex was also significantly increased in HSD group (*P* < 0.05, *P* < 0.001) and in Wogonin group (*P* < 0.01, *P* < 0.001) (Fig. 8C).

FISH assay was used to observe the distribution and relative content of bacteria or bacterial DNA fragments in the brain of mice in each group. The results showed that the model group had significantly increased bacteria-specific fluorescence signal values in the hippocampus and cortex comparing with the control group (*P* < 0.001, *P* < 0.001). Compared with the model group, the bacteria specific fluorescence signal values in the hippocampus and cortex of the donepezil group and HSD group were both significantly decreased (*P* < 0.001, *P* < 0.001; *P* < 0.001, *P* < 0.01). In the Wogonin group, there was no significant difference in the value of bacteria-specific fluorescence signal in the hippocampus, but it was significantly reduced in cortex (*P* < 0.001) (Fig. 8D). Immunofluorescence staining was used to observe the distribution and relative content of LPS in the brain of mice in each group. The results showed that the model group had significantly increased LPS expression in the hippocampus and cortex comparing with the control group (*P* < 0.001, *P* < 0.01). Compared with the model group, the donepezil group had significantly reduced LPS expression in the hippocampus and cortex (*P* < 0.001, *P* < 0.01), which was also observed in HSD group (*P* < 0.001, *P* < 0.01) and Wogonin group (*P* < 0.01, *P* < 0.001) (Fig. 8E).

### HSD regulates the structure of intestinal microbiota in samp8 mice

**Fig. 9.**
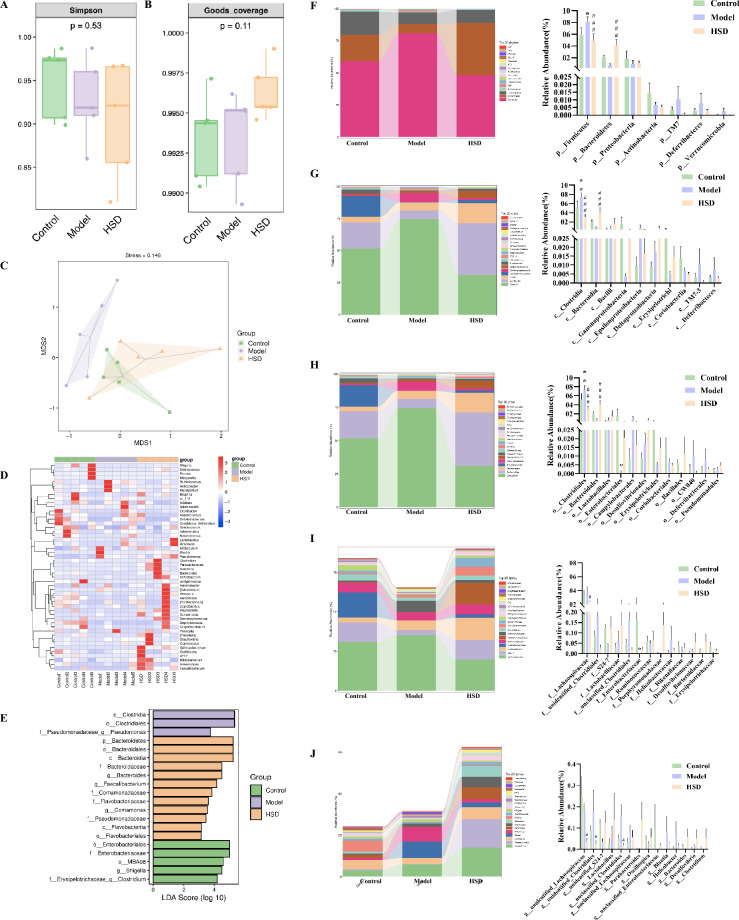
16S rRNA analysis of intestinal microbiota. **A** Simpson value of alpha diversity index; **B** Coverage value of alpha diversity index; **C** NMDS analysis of beta diversity; **D** Genus-level species composition heatmap of species clustering; **E** Lefse analysis of bacterial species differences among groups; **F** Species composition and statistical analysis at phylum level; **G** Species composition and statistical analysis at class level; **H** Species composition and statistical analysis at the order level; **I** Species composition and statistical analysis at the family level; **J** Species composition and statistical analysis at the genus level. The data are expressed as Mean ± SEM (*n* = 5). *: model group compared with the control group, #: HSD group compared with the model group, *^,#^*P* < 0.05, **^,##^*P* < 0.01, ***^,###^*P* < 0.001.*model group compared with the control group, ^#^HSD group compared with the model group

Alpha diversity showed that the Simpson and coverage values of HSD group were increased comparing with the model group with no significant difference (Fig. 9A, B). NMDS analysis of Beta diversity showed that the microbiota structure of the control group and the model group was relatively independent, while there was overlap between the control group and the HSD group, indicating that the microbiota composition of the HSD group was more similar to that of the control group (Fig. 9C). The heat map showed the genus-level composition of the intestinal bacterial clusters of mice in each group. The red blocks represented the abundance of genus was higher than others and the blue blocks represented lower (Fig. 9D). Lefse analysis showed the difference in bacterial species among groups. In the control group, the species difference bacteria included *enterobacteriales*, *Enterobacteriaceae*, MBAO8, *shigella*, *clostridium*; In the model group, the species difference bacteria included *clostridia*, *clostridiaies*, *pseudomonas*; The species difference bacteria in HSD group included *bacteroidetes*, *bacteroidales*, *bacteroidia*, *bacteroidaceae*, *bacteroides*, *faecalibacterium*, *comamonadaceae*, *flavobacteriaceae*, *comamonas*, *Pseudomonadaceae*, *flavobacteria*, *flavobacteriales* (Fig. 9E). The composition of intestinal microbiota showed significant differences in the abundance of ASVs/OTU sequences among different groups at the phylum, class, order, family, and genus levels. The results showed that the abundance of *Firmicutes* was significantly increased in the model group compared with the control group at the phylum level (*P* < 0.05). Compared with the model group, the HSD group had a significant reduction in the abundance of *Firmicutes* (*P* < 0.001) and a significant increase in the abundance of *Bacteroidetes* (*P* < 0.001) (Fig. 9F). At the class level, compared with the control group, the abundance of *Clostridia* in the model group was significantly increased (*P* < 0.01). Compared with the model group, the HSD group had a significant reduction in the abundance of *Clostridia* (*P* < 0.001) and a significant increase in the abundance of *Bacteroidia* (*P* < 0.001) (Fig. 9G). At the order level, the abundance of *Clostridiales* in the model group was significantly increased compared with the control group (*P* < 0.01), and the abundance of *Enterobacteriales* was significantly decreased (*P* < 0.05). Compared with the model group, the HSD group had a significantly lower abundance of *Clostridiales* (*P* < 0.001) and a significantly higher abundance of *Bacteroidales* (*P* < 0.001) (Fig. 9H). At the family level, compared with the control group, the abundance of *Lachnospiraceae* in the model group was significantly reduced (*P* < 0.05). Compared with the model group, the abundance of *Enterobacteriaceae* in HSD group was significantly decreased (*P* < 0.05) (Fig. 9I). At the genus level, compared with the model group, the abundance of *unidentified_Lachnospiraceae*, *unidentified_Clostridiales*, and *unclassified_Clostridiales* was significantly reduced (*P* < 0.05) (Fig. 9J).

## Discussion

The compounds identification of drug-containing serum showed that Wogonin was one of the components of HSD into blood. It was reported that Wogonin is a potential drug to treat AD [28]. In this study, pharmacodynamic experiments were first performed. Behavioral experiments showed that both HSD and Wogonin improved the learning and memory ability of SAMP8 mice. HSD and Wogonin reduced the phosphorylation level of tau protein and Aβ deposition in the brain of SAMP8 mice, indicating that HSD and Wogonin can alleviate the AD pathology of SAMP8 mice. The results of H&E staining showed that HSD and Wogonin reduced the neuronal loss and alleviate the pathological changes of neuronal morphology of SAMP8 mice. Transmission electron microscope observation showed that the synaptic structure and synaptic gap in the hippocampus of HSD and Wogonin-treated mice were clear indicating that HSD and Wogonin had protective effect on neuronal structure damage. Therefore, it is confirmed the therapeutic effect of HSD and its components in the treatment of AD.

In order to explore the mechanism of HSD and Wogonin in the improvement of AD, proteomic analysis was performed in hippocampus of mice. Our proteomics study identified FG as a potential therapeutic target of HSD in SAMP8 mice. FG deposition serves as a marker of BBB dysfunction and central nervous system pathology [29]. FG is a kind of glycoprotein synthesized and secreted by hepatocytes which includes α, β and γ polypeptide chains which forms a stable structure of cross-linked fibrin chain with integrated red blood cells and platelets, promoting the stability and proliferation of endothelial cells. Aβ pathological products will be deposited in the brain parenchyma and blood vessels which once enter the blood will induce thrombin to convert fibrinogen into insoluble fibrin, then co-deposit with Aβ to form fibrin clots that are difficult to degrade [30]. Normally, FG is excluded from the brain by the BBB [31, 32]. The disruption of BBB allows FG to enter brain parenchyma, eliciting immune reactivity and response [33]. Research showed that Aβ-FG interaction can lead to neurovascular injury, BBB dysfunction, and increased permeability, thereby accelerating the persistent deposition of FG in brain tissue [34]. Therefore, FG in blood vessels is closely related to AD. In this study, proteomics analysis and experimental verification showed that the β chain and γ chain of FG were significantly increased in the brain of SAMP8 mice which were reduced by HSD and Wogonin. We further found that both FGB and FGG colocalize with Aβ in the hippocampus and cortex which suggested that FG may affect AD pathology by co-or interacting with Aβ.

Importantly, FG is critical in the inflammatory system of AD [35]. After the destruction of BBB, fibrinogen and fibrinogen oligomer deposition in the central nervous system further lead to neuroinflammation by activating glial cells, causing the destruction and loss of nerve dendritic spines [36]. Meanwhile, glial cell inflammation also accelerates the permeability of BBB, leading to FG leakage [37], causing neurovascular injury and neuronal dysfunction and accelerating AD neuropathology. The results in our study showed that FGB and FGG were co-located with microglia marker Iba-1, indicating that the abnormal activation of microglia may be related to FG which is similar to the previous report [36]. Moreover, FG has a strong correlation with astrocytes. FG binds to astrocytes at the site of BBB disruption to induce ROS release and the expression of proinflammatory factors, which induce neuronal death [38]. The results in our present study showed that FGB and FGG were co-localized with GFAP, an astrocyte marker, indicating that abnormal astrocyte activation may be in connection with FG, and the inhibition of astrocyte activation by HSD and Wogonin may be related to the reduction of FG expression. Oligodendrocytes are the only source of myelin formation and generate multiple myelin sheathe to wrap axons and ensure the rapid and efficient conduction of action potentials along axons. Myelin damage is present in AD, and 3×Tg and 5×FAD transgenic AD mice show significant region-specific changes in myelin before the appearance of characteristic pathological products [39, 40]. FG deposits in the central nervous system after disruption of the BBB which induce encephalitic adaptive immune responses and recruitment of peripheral macrophages to the central nervous system, leading to demyelination [41]. At the same time, FG inhibits neural repair, which is achieved by preventing the differentiation of oligodendrocyte progenitors to myelinated oligodendrocytes [42]. In this study, HSD and Wogonin increased the expression of MBP in SAMP8 mice, indicating a protective effect of HSD on oligodendrocytes.

In order to further explore the protective effects of HSD on the BBB, the blood vessel related markers were detected. ZO-1 and Occludin are essential components of tight junctions between cells [43]. During the occurrence and development of AD, the decreased protein levels of ZO-1 and Occludin in brain will lead to structural disorder of vascular endothelial cells, cerebrovascular injury, increased permeability of BBB, and accelerated accumulation of Aβ [44]. This study showed that the protein levels of ZO-1 and Occludin in brain of SAMP8 mice were significantly reduced. Transmission electron microscopy showed that the basement membrane of microvessels in the hippocampus of SAMP8 mice was broken and blurred, which may be an important reason for the destruction of BBB. Wogonin has been reported to improve functional neuroprotection in rats with acute cerebral ischemia, which was achieved by promoting angiogenesis [45], modulated Aβ-induced toxicity [46] and had the potential for clinical treatment of AD [47]. Furthermore, in this study, it is found that HSD and Wogonin significantly increased the protein levels of ZO-1 and Occludin in brain, and reduced the damage of microvascular structure, suggesting that the treatment of AD with HSD and Wogonin is associated with improvement of the BBB.

AD is accompanied by disruption of the gut microbiota [48]. In this study, the bacteria structure of SAMP8 mice converged to a healthy level after HSD treatment. HSD reduced the abundance of *Firmicutes*, *Clostridia*, *Clostridiales* and *Enterobacteriaceae*, increased the abundance of *Bacteroidetes*, *Bacteroidia* and *Bacteroidales*, suggesting that HSD is able to regulate the intestinal microbiota structure in SAMP8 mice. It is known that intestinal microbiota contains a large amount of LPS, which is the cell wall composition of Gram-negative bacteria [49]. The unbalanced intestinal flora will destroy the integrity of the intestinal barrier and destroy the intestinal function [50, 51]. Studies have shown that bacterial cell wall component LPS accumulates in the brain parenchyma of AD patients [52–54]. The increase of LPS in the brain may be in connection with the disruption of the intestinal barrier and the invasion of LPS from the circulatory system to the central system, or may be due to the presence of microflora in the brain nervous system [54]. Interestingly, researchers first found that the number of bacteria in brain of AD patients increased compared with that of patients with normal cognitive function by performing 16S rRNA next-generation sequencing on postmortem brain slices of AD patients in 2017 [55]. In this study, it was found that the levels of bacteria/bacterial DNA fragments and LPS in brain of mice in the model group were significantly increased. We considered that the sources of bacteria/bacterial DNA fragments and LPS in the mice brain detected in this study may include two aspects, one is the bacteria and cell wall components in the brain, the other one is the bacteria and LPS components in the gut which enter the brain in the way of the destruction of the gut-brain barrier and the blood circulation. HSD and Wogonin reduced the level of bacteria/DNA fragments and LPS in the brain of SAMP8 mice, which may be related to the regulation of BBB function. This study also supports the neuroprotective effects of plant-derived bioactive compounds. Studies have reported that in addition to plant-derived bioactive compounds, there are also bioactive compounds from other sources, such as Marine sources [56], that can exert neuroprotective effects, which together bring hope to the treatment of AD.

## Conclusion

**Fig. 10:**
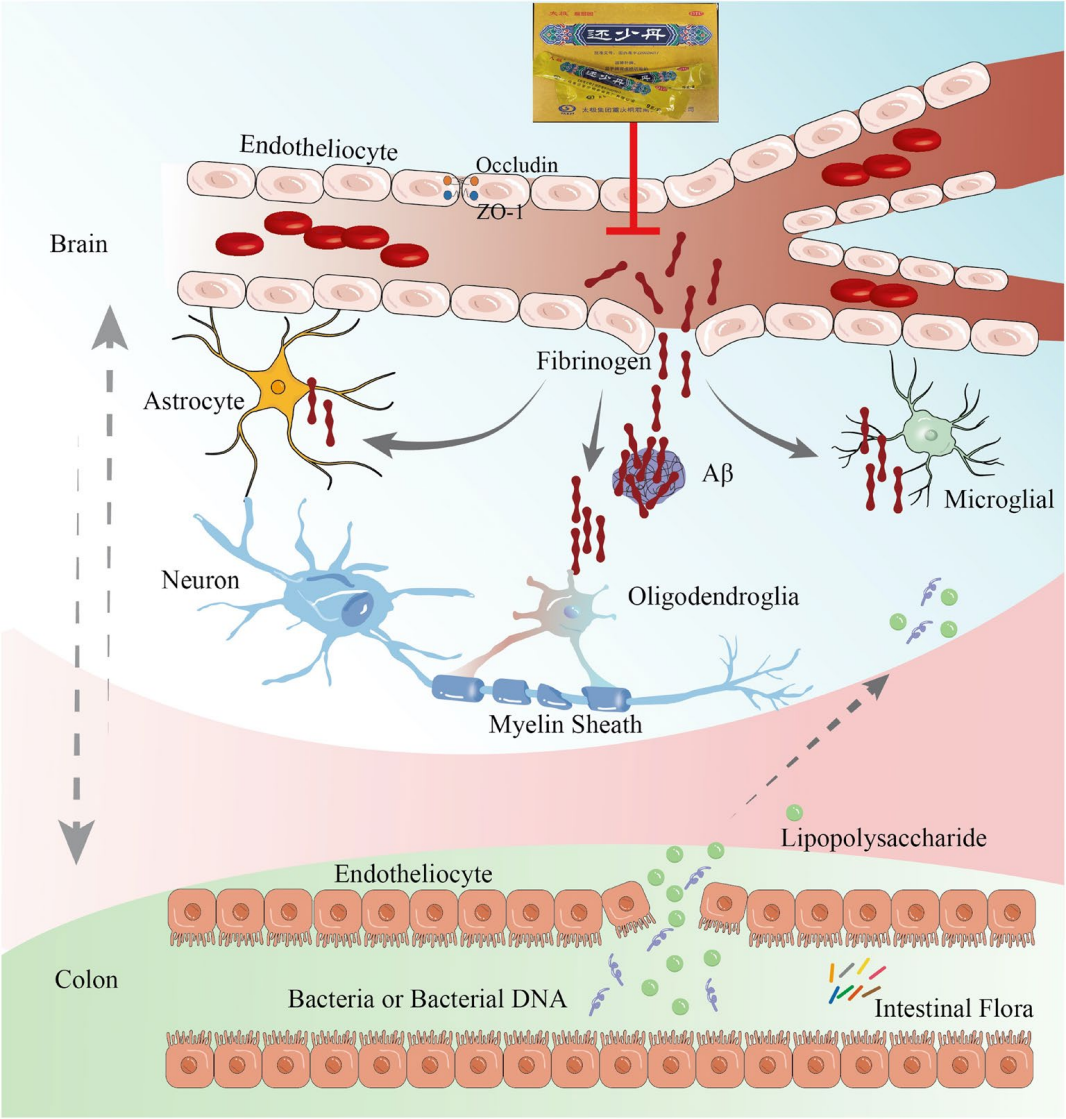
The potential mechanism of the treatment of HSD in AD. HSD protected the BBB of AD mice, the underlying mechanism of which may be due to the regulation of intestinal microbiota and the inhibition of FG and its effect on glia cells in the brain

HSD and its compound in drug-containing serum protected the BBB in AD mice, the underlying mechanism of which may be related to inhibit the levels of FG and interact on glia cells in the brain, and by modulating the structure of intestinal microbiota and improving the blood–brain barrier function (Fig. 10). However, this study only confirmed the pharmacological effect and related pathological process of HSD and Wogonin, but the mechanism by which HSD and its components reducing FG deposition and inhibiting glial cell activation has not been deeply studied. Moreover, the specific types of bacteria/bacterial DNA in the brain of SAMP8 mice reduced by HSD are still unclear. In the future research, it will conduct in-depth mechanism exploration basing on this study, and verify the pharmacological effect and mechanism of HSD, so as to provide scientific basis and theoretical support for the rational and scientific application of HSD in clinical practice, provide research basis for the screening of effective components of anti-AD, and provide ideas and reference for the research and development of new anti-AD drugs.

## Supplementary information

Supplementary Material 1

Supplementary Material 2

Supplementary Material 3

## Data Availability

The original contributions presented in the study are all included in the article/Supplementary Material, further inquiries can be directed to the corresponding author.

## Abbreviations

AD: Alzheimer’s disease
Aβ: Amyloid β-protein
HSD: Huanshaodan
FG: Fibrinogen
FGB: Fibrinogen beta chain
FGG: Fibrinogen gamma chain
Iba-1: Ionized calcium binding adapter molecule 1
GFAP: Glial fibrillary acidic protein
ECM: Extracellular matrix
TEM: Transmission electron microscopy
LPS: Lipopolysaccharide
MBP: Myelin basic protein
BBB: Blood-brain barrier
