# Supplementary material for: Proteomic analysis and experimental validation reveal the blood–brain barrier protective of Huanshaodan in the treatment of SAMP8 mouse model of Alzheimer’s disease
Source: Chin Med. 2024 Oct 5;19:137. doi: 10.1186/s13020-024-01016-7 (PMC11456246; doi:10.1186/s13020-024-01016-7)
Supplement: Supplementary file 1 — Supplementary Material 1 [file 13020_2024_1016_MOESM1_ESM.pptx]

## Slide 1
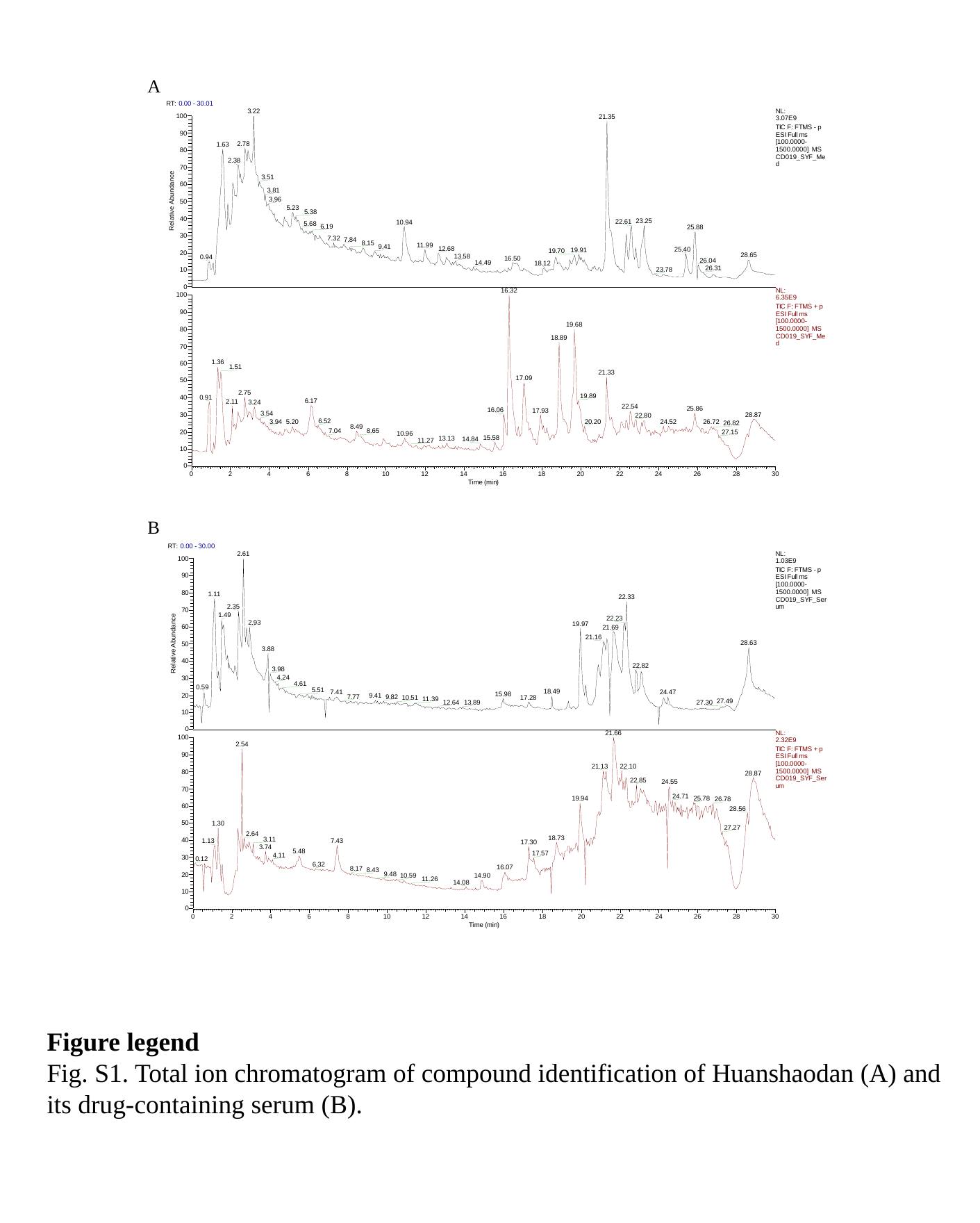

A
B
Figure legend
Fig. S1. Total ion chromatogram of compound identification of Huanshaodan (A) and its drug-containing serum (B).
