# Supplementary figures and images for: Proteomic analysis and experimental validation reveal the blood–brain barrier protective of Huanshaodan in the treatment of SAMP8 mouse model of Alzheimer’s disease
Source: Chin Med. 2024 Oct 5;19:137. doi: 10.1186/s13020-024-01016-7 (PMC11456246; doi:10.1186/s13020-024-01016-7)

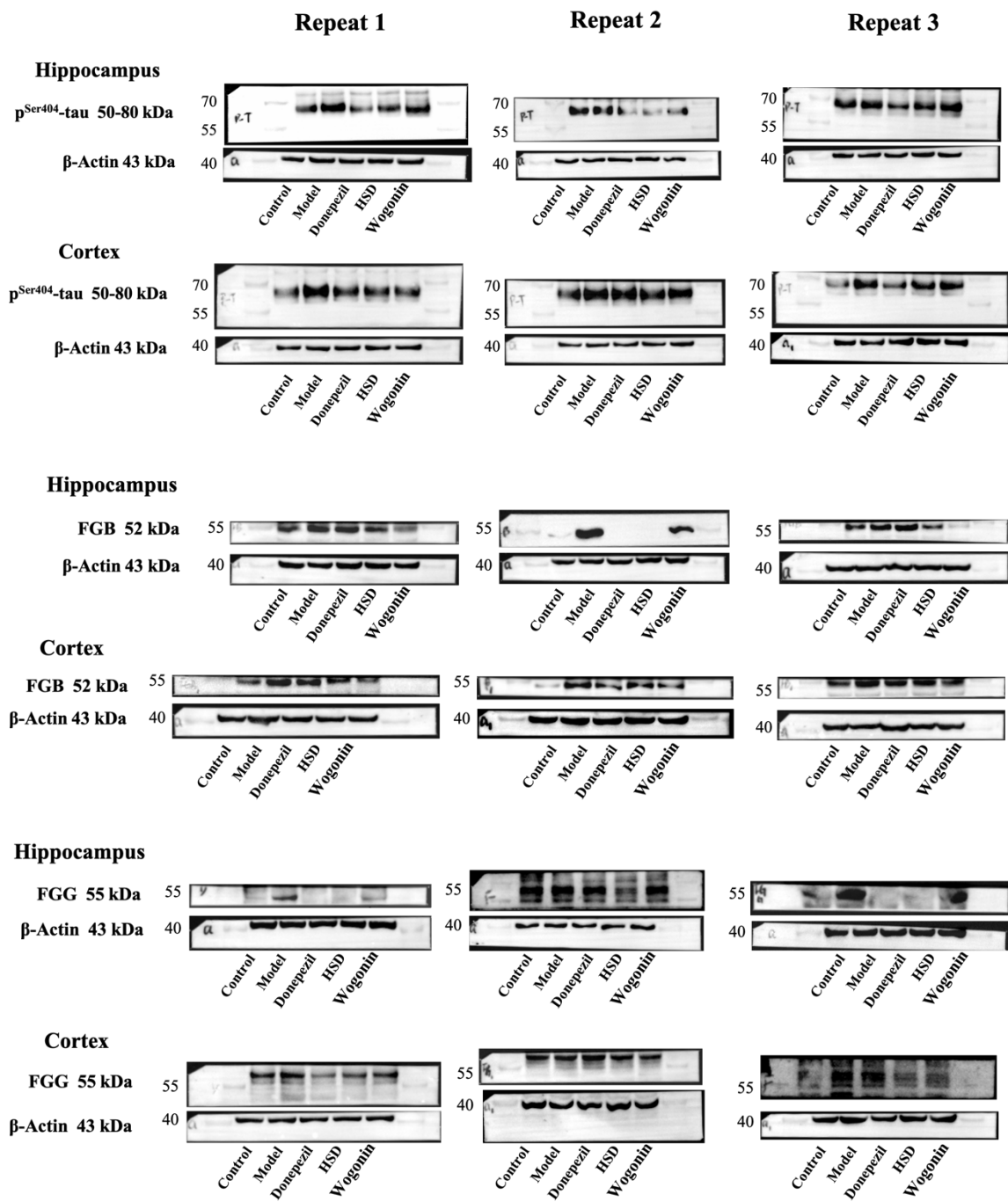

## Hippocampus

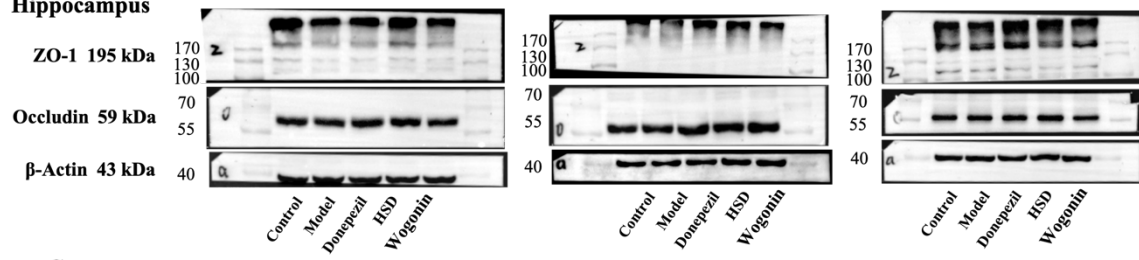

## Cortex

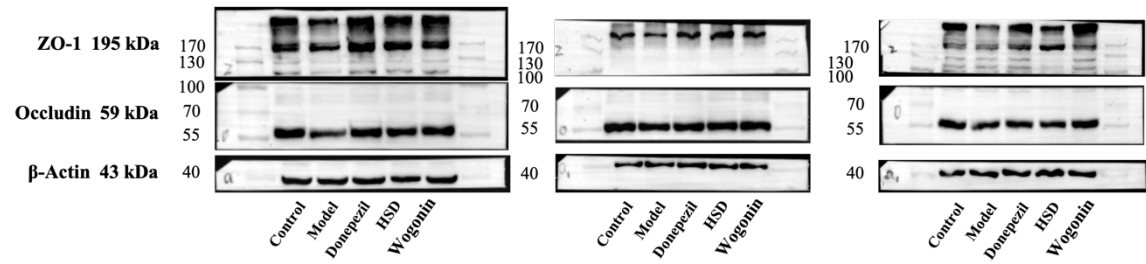

Supplement: Supplementary file 2 — Supplementary Material 2 [file 13020_2024_1016_MOESM2_ESM.pdf]
