# Supplementary material for: Proteomic analysis and experimental validation reveal the blood–brain barrier protective of Huanshaodan in the treatment of SAMP8 mouse model of Alzheimer’s disease
Source: Chin Med. 2024 Oct 5;19:137. doi: 10.1186/s13020-024-01016-7 (PMC11456246; doi:10.1186/s13020-024-01016-7)
Supplement: Supplementary file 3 — Supplementary Material 3 [file 13020_2024_1016_MOESM3_ESM.docx]

**Tables**

Table S1 Compounds in the Huanshaodan

| Number /Name/ Formula | Structure | Number /Name/ Formula | Structure | Number /Name/ Formula | Structure |
| --- | --- | --- | --- | --- | --- |
| 1. Wogonin   C16 H12 O5 |  | 1. Vanillin   C8 H8 O3 |  | 1. Diosmetin   C16 H12 O6 |  |
| 1. Kaempferol   C15 H10 O6 |  | 1. Ferulic acid   C10 H10 O4 |  | 1. Geniposidic acid   C16 H22 O10 |  |
| 1. Carvone   C10 H14 O |  | 1. Cantharidin   C10 H12 O4 |  | 1. Ursolic acid   C30 H48 O3 |  |
| 1. Isoliquiritigenin   C15 H12 O4 |  | 1. Psoralen   C11 H6 O3 |  | 1. 4-Coumaric acid   C9 H8 O3 |  |
| 1. Zerumbone   C15 H22 O |  | 1. Piceatannol   C14 H12 O4 |  | 1. Genistein   C15 H10 O5 |  |
| 1. Loganin   C17 H26 O10 |  | 1. β-Asarone   C12 H16 O3 |  | 1. Scoparone   C11 H10 O4 |  |
| 1. Coumarin   C9 H6 O2 |  | 1. Nootkatone   C15 H22 O |  | 1. Methyl cinnamate   C10 H10 O2 |  |
| 1. Formononetin   C16 H12 O4 |  | 1. 5-Hydroxymethyl-   2-furaldehyde  C6 H6 O3 |  | 1. trans-Anethole   C10 H12 O |  |
| 1. Asiatic acid   C30 H48 O5 |  | 1. Rubiadin   C15 H10 O4 |  | 1. α-Linolenic acid   C18 H30 O2 |  |
| 1. Scopoletin   C10 H8 O4 |  | 1. Schisandrin   C24 H32 O7 |  | 1. Erucic acid   C22 H42 O2 | 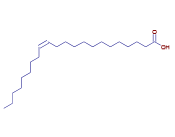 |

Table S2 Compounds in the Huanshaodan drug-containing serum

| Number /Name/ Formula | Structure | Number /Name/ Formula | Structure | Number /Name/ Formula | Structure |
| --- | --- | --- | --- | --- | --- |
| 1. Wogonin  C16 H12 O5 |  | 22. Tetradecanedioic acid  C14 H26 O4 |  | 42. Erucic acid  C22 H42 O2 |  |
| 2. N,N'-Dicyclohexylurea  C13 H24 N2 O |  | 23. Indole-3-lactic acid  C11 H11 N O3 |  | 43. 2,2,6,6-Tetramethyl-4-piperidinol  C9 H19 N O |  |
| 3. Triphenylphosphine oxide  C18 H15 O P |  | 24. Hexadecanamide  C16 H33 N O |  | 44. Docosahexaenoic acid  C22 H32 O2 |  |
| 4. Indole-3-acrylic acid  C11 H9 N O2 |  | 25. N-Acetyl-DL-tryptophan  C13 H14 N2 O3 |  | 45. 4,4-Diphenylmethane diisocyanate  C15 H10 N2 O2 |  |
| 5. L-Phenylalanine  C9 H11 N O2 |  | 26. Isoleucine  C6 H13 N O2 |  | 46. Pantothenic acid  C9 H17 N O5 |  |
| 6. Bis (4-ethylbenzylidene)sorbitol  C24 H30 O6 |  | 27. Phloroglucinol  C6 H6 O3 |  | 47. Prostaglandin F2α 1-11-lactone  C20 H32 O4 |  |
| 7. Palmitic acid  C16 H32 O2 |  | 28. Uric acid  C5 H4 N4 O3 |  | 48. Stearamide  C18 H37 N O |  |
| 8. trans-Petroselinic acid  C18 H34 O2 |  | 29. Docosapentaenoic acid  C22 H34 O2 |  | 49. Glycitein  C16 H12 O5 |  |
| 9. Linoleic acid  C18 H32 O2 |  | 30. 2,4-Dimethylbenzaldehyde  C9 H10 O |  | 50. 7-Hydroxycoumarine  C9 H6 O3 |  |
| 10. Palmitoylcarnitine  C23 H45 N O4 |  | 31. 11(Z),14(Z)-Eicosadienoic acid  C20 H36 O2 |  | 51. 13(S)-HOTrE  C18 H30 O3 |  |
| 11. Hexanoylcarnitine  C13 H25 N O4 |  | 32. Palmitoleic acid  C16 H30 O2 |  | 52. L-(-)-Methionine  C5 H11 N O2 S |  |
| 12. 3,5-di-tert-Butyl-4-hydroxybenzaldehyde  C15 H22 O2 |  | 33. Azelaic acid  C9 H16 O4 |  | 53. Cuminaldehyde  C10 H12 O |  |
| 13. Docosahexaenoic acid  C22 H32 O2 |  | 34. 4-Indolecarbaldehyde  C9 H7 N O |  | 54. 17α-Hydroxypregnenolone  C21 H32 O3 |  |
| 14. Arachidonic acid  C20 H32 O2 |  | 35. Hexanoylglycine  C8 H15 N O3 |  | 55. 2-Amino-1,3,4-octadecanetriol  C18 H39 N O3 |  |
| 15. Deoxycholic acid  C24 H40 O4 |  | 36. 3-Hydroxydecanoic acid  C10 H20 O3 |  | 56. 2,5-di-tert-Butylhydroquinone  C14 H22 O2 |  |
| 16. DL-Tryptophan  C11 H12 N2 O2 |  | 37. Adrenic acid  C22 H36 O2 |  | 57. Scopoletin  C10 H8 O4 |  |
| 17. Oleic acid alkyne  C18 H30 O2 |  | 38. 2,2'-Methylenebis(4-methyl-6-tert-  butylphenol)  C23 H32 O2 |  | 58. (15Z)-9,12,13-Trihydroxy-15-octadecenoic acid  C18 H34 O5 |  |
| 18. 8Z,11Z,14Z-Eicosatrienoic acid  C20 H34 O2 |  | 39. Methyl indole-3-acetate  C11 H11 N O2 |  | 59. Quinoline  C9 H7 N |  |
| 19. 11,12-Epoxy-(5Z,8Z,11Z)-icosatrienoic  acid  C20 H32 O3 |  | 40. Cinnamoylglycine  C11 H11 N O3 |  | 60. Flavone  C15 H10 O2 |  |
| 20. Stearic acid  C18 H36 O2 |  | 41. 14(Z)-Eicosenoic acid  C20 H38 O2 |  | 61. 4-Hydroxybenzaldehyde  C7 H6 O2 |  |
| 21. cis-5,8,11,14,17-Eicosapentaenoic acid  C20 H30 O2 |  |  |  |  |  |

Table S3 Differential proteins identified by proteomic analysis

| **No.** | **Gene number** | **Gene name** | **Protein name** | **Regulated by HSD** |
| --- | --- | --- | --- | --- |
| 1 | Q9JK53 | Prelp | Prolargin | Down-regulated |
| 2 | P11087 | Col1a1 | Collagen alpha-1(I) chain | Down-regulated |
| 3 | Q01149 | Col1a2 | Collagen alpha-2(I) chain | Down-regulated |
| 4 | Q8K0E8 | Fgb | Fibrinogen beta chain | Down-regulated |
| 5 | E9QAE3 | Btaf1 | B-TFIID TATA-box-binding protein-associated factor 1 | Down-regulated |
| 6 | Q99JT9 | Adi1 | 1,2-dihydroxy-3-keto-5-methylthiopentene dioxygenase | Down-regulated |
| 7 | A2A9V7 | Slc25a19 | Mitochondrial thiamine pyrophosphate carrier (Fragment) | Up-regulated |
| 8 | Q60847 | Col12a1 | Collagen alpha-1(XII) chain | Down-regulated |
| 9 | Q8VCM7 | Fgg | Fibrinogen gamma chain | Down-regulated |
| 10 | F7CD65 | Epn2 | Epsin-2 (Fragment) | Up-regulated |
| 11 | E9Q7M2 | Tsc22d2 | TSC22 domain family, member 2 | Up-regulated |
| 12 | Q01339 | Apoh | Beta-2-glycoprotein 1 | Down-regulated |
